# Dynamic coherent backscattering mirror

**DOI:** 10.1063/1.4941832

**Published:** 2016-02-08

**Authors:** I. Zeylikovich, M. Xu

**Affiliations:** Physics Department, Fairfield University, Fairfield, CT 06824, USA

## Abstract

The phase of multiply scattered light has recently attracted considerable interest. Coherent backscattering is a striking phenomenon of multiple scattered light in which the coherence of light survives multiple scattering in a random medium and is observable in the *direction* space as an enhancement of the intensity of backscattered light within a cone around the retroreflection direction. Reciprocity also leads to enhancement of backscattering light in the *spatial* space. The random medium behaves as a reciprocity mirror which robustly converts a diverging incident beam into a converging backscattering one focusing at a conjugate spot in space. Here we first analyze theoretically this coherent backscattering mirror (CBM) phenomenon and then demonstrate the capability of CBM compensating and correcting both static and dynamic phase distortions occurring along the optical path. CBM may offer novel approaches for high speed dynamic phase corrections in optical systems and find applications in sensing and navigation.

Recent development in adaptive optics and wave-front shaping techniques has highlighted the significance of the phase of multiply scattered light and demonstrated the manipulation of the phase can overcome multiple scattering and focus the multiply scattered light across or inside a turbid medium (see the recent reviews^1–3^ and references within). The basic principle is through either explicit^4–6^ or implicit^7^ phase conjugation of the scattered wave. Another well-known phenomenon of multiple scattered light in which the coherence of light survives multiple scattering is coherent backscattering.^8–11^ Since the first experimental observation of coherent backscattering from colloidal suspensions,^8,9,12^ the phenomenon has been studied in a variety of different media such as powders,^13,14^ biological tissues,^15^ photonic crystals,^16^ cold atom gases,^17,18^ and liquid crystals.^19^ Coherent backscattering is responsible for the so called weak localization phenomenon, which is the precursor of Anderson localization.^20,21^ In a conventional coherent backscattering experiment, a collimated beam is reflected by a turbid medium and the backscattered light is found in the *direction* space to enhance within a cone around the retroreflection direction, originating from the constructive interference between the light waves propagating along a pair of time-reversal trajectories (see Fig. 1a).

Reciprocity^22,23^ also leads to enhancement of backscattering light in the *spatial* space. When a random medium acts as a coherent backscattering mirror (CBM), a diverging beam is reflected by the turbid medium and converges to a conjugate spot (to the waist of the incident beam) in the *spatial* space whose intensity is enhanced due to the constructive interference between the light waves propagating along a pair of reciprocal trajectories (see Fig. 1b). This is the consequence of the well-known reciprocity relation of electromagnetic waves.^22,23^ In the case of quasinormal incidence and emergence, this symmetry ensures the electromagnetic wave in the helicity-preserving polarization channel experiences identical phase delay along the forward or time-reversal paths inside the scattering medium. This enhancement of backscattered light in the spatial space behaves as pseudo phase conjugation and has been used in correcting static phase distortions.^24^

In this Letter we first analyze theoretically this coherent backscattering mirror phenomenon and then demonstrate the capability of CBM compensating and correcting both static and dynamic phase distortions occurring along the optical path. Potential applications of CBM for high speed dynamic phase corrections in optical systems, sensing and navigation are discussed at the end.

Consider a Gaussian beam normally incident upon a scattering medium (see Figs. 1b and 2a). The optical path difference between the pair of trajectories *P*_1_*P*_2_*Q* and *P*_2_*P*_1_*Q* can be written as $q_{⊥}\cdot \rho +q_{⊥}^{\prime }\cdot \rho$ where **q**_⊥_ ≡ *k****ρ***_*d*_/*L*, $q_{⊥}^{\prime }\equiv k\overset{¯}{\rho }\left(L-R\right)/LR$, ***ρ*** ≡ ***ρ***_2_ − ***ρ***_1_, $\overset{¯}{\rho }=\left(\rho_{2}+\rho_{1}\right)/2$, *k* = 2*π*/λ with λ the wavelength in vacuum, ***ρ***_1,2_ and ***ρ***_*d*_ are the lateral positions on the surface of and the observation plane at a distance, *L*, above the scattering medium, respectively, and *R* is the radius of curvature of the incident beam. The $q_{⊥}^{\prime }\cdot \rho$ term originates from the placement of the observation plane away from the conjugate position *L* = *R*. The two rays *P*_1_*P*_2_*Q* and *P*_2_*P*_1_*Q* interfere and generate an enhanced spot on the observation plane in the *spatial* space . The enhancement spots, in 3D, merge into an enhancement spindle peaked at and normal to the conjugate plane.

The distribution of the intensity of backscattering light on the observation plane subject to the incident beam of unit power is given by^25^:

$$I_{\mathrm{cbs}}\left(\rho_{d}\right)=\int d^{2}\rho \left[1+\exp\left(-\frac{\rho^{2}}{2w^{\prime 2}}\right)\cos\left(q_{⊥}\cdot \rho \right)\right]I\left(\rho_{1},\rho_{2}\right)$$ (1)

where *I*(***ρ***_1_, ***ρ***_2_) is the propagator for light normally incident at ***ρ***_1_, propagating inside the scattering medium, and emerging normally at ***ρ***_2_, and *w*′ is the effective beam spot size (radius) on the surface given by $w/\sqrt{1+k^{2}{\left(L-R\right)}^{2}w^{4}/4L^{2}R^{2}}$. The value of *w*′ reduces to the beam width *w* when observed on the conjugate plane. The coherent backscattering spot takes a simple form in terms of the light propagator, *I*(***κ***), in the spatial frequency domain and can be rewritten as

$$I_{\mathrm{cbs}}\left(\rho_{d}\right)=I_{\mathrm{incoh}}+\int d^{2}\kappa I\left(\kappa \right)g\left(q_{⊥}-\kappa \right)$$ (2)

from Eq. (1) where ***κ*** is the spatial frequency, *I*_incoh_ ≡ ∫*d*^2^***ρ****I*(***ρ***) is the incoherent background, and

$$g\left(q\right)=\frac{1}{2\pi }w^{\prime 2}\exp\left(-\frac{w^{\prime 2}q^{2}}{2}\right)$$

is the *effective* spatial frequency profile of the incident Gaussian beam. The diffusion approximation to radiative transfer has been applied successfully to model *I*(***κ***) in coherent backscattering of light.^10,26^

The enhanced peak is centered at **q**_⊥_ = ***ρ***_*d*_ = 0. The profile of the enhanced spot at ***ρ***_*d*_ = *L****θ*** or **q**_⊥_ = *k****θ*** on the observation plane is determined by the convolution of the spatial frequency spreads of the light propagator inside the scattering medium and the incident beam on the interface plane. The former has an angular spread Δ*θ_s_* = λ/3*πl_t_* due to scattering under the diffusion approximation.^10^ The latter has an angular spread $\Delta \theta_{b}=\sqrt{2\log2}\lambda /\pi w^{\prime }$ which *shrinks* with the effective beam spot size *w*′. The sharpest enhanced spot is observed on the conjugate plane. The enhancement effect survives over a range of *L*, forming a enhancement spindle in 3D along the axis normal to the conjugate plane (see Fig. 2b). When *l_t_* ≪ *w*′, CBM reduces to the conventional CBS configuration with the enhancement factor *E* = 2 and full width at half maximum (FWHM) 2*θ*_1/2_ = Δ*θ_s_*. When *l_t_* ≫ *w*′, the size of the enhanced spot scales with 2*θ*_1/2_ = Δ*θ_b_* and the enhancement factor in this limit approaches *E* = 1 + 2*πw*^′2^*I*(***ρ*** = 0)/*I*_incoh_. In the latter limit, the enhanced spot size reduces to $2L\theta_{1/2}=\sqrt{2\log2}w_{0}$ on the conjugate plane, identical to the FWHM of the incident beam at its waist when *L* ≫ *z*_0_.

Figure 3 displays the enhancement factor *E* = *I*_cbs_(***ρ***_*d*_ = 0)/*I*_incoh_ and the normalized FWHM, 2*kl_t_θ*_1/2_, of the enhanced spot on the conjugate plane computed from the diffusion approximation to radiative transfer, the diffusion approximation with snake photon correction,^25,27^ and Monte Carlo simulations^28^ of photons inside an index-matched semi-infinite scattering medium of Rayleigh scatterers or Mie scatterers (polystyrene spheres of diameter 1.5*μm* in water, *g* = 0.92 at λ = 0.515*μm*), respectively. The diffusion approximation, in particular, with the snake photon correction, describes well the enhancement profile.

Figure 4 shows the excellent agreement between the theoretical model (2) and the experimental data for light backscattering by a suspension of polystyrene spheres (size: 0.49*μm*) in water (slab thickness 8mm),.^25^ The theoretical model with the *only free parameter* *l_t_* was fitted to the experimental data for volume concentration of (a) 1% and (b) 0.5% polystyrene sphere suspensions, yielding 0.279*mm* and 0.534*mm*, respectively. Their values agree well with the expected *l_t_* predicted by Mie theory (0.270*mm* and 0.540*mm*, respectively).

CBM can automatically compensate and correct phase distortion occurring on the optical path. Such capability of CBM was demonstrated following the setup given in Fig 5. In the first experiment, a plastic cylinder having 1 mm outer and 0.5 mm internal diameter was placed perpendicular to the incident laser beam in front of a cuvette containing Intralipid-10% suspension serving as CBM in which the plastic cylinder introduced significant phase distortions to the incident wavefront. In a second experiment a thin scattering paper was used as a phase distorting medium to demonstrate that the CBM could compensate for distortions in the optical system caused by weak light scattering. In both cases, the pattern of illumination on the suspension surface was severely deformed. Figure 6 illustrates the point spread function (PSF) images obtained with CBM compared to the case without any phase distortion in the optical path. The width of the PSF stays relatively unchanged whereas the intensity of the CBS peak decreases with the introduction of phase distortion. The PSF images obtained with a plane mirror in place of the CBM in these two experiments were severely distorted. The reduction in the enhancement is caused by the increase in the background intensity owing to stray light produced by (weak) scattering in the phase distortion medium. Such scattering in the phase distortion medium also increases the area of illumination on the suspension surface, resulting in slightly narrower FWHM for the PSF, more appreciably, in the case of a thin scattering paper. The CBM performs as well in correcting the dynamic phase distortions as in correcting static ones. The PSF image obtained from a fast moving phase aberrator (the same plastic cylinder) is almost identical to that from the static plastic cylinder and has slightly higher enhancement and narrower FWHM due to its effective larger illumination area.

The static and dynamic distortion correction exhibited by CBM can be attributed to the automatic phase compensation of the reciprocal beams when light is reflected by CBM in the helicity-preserved polarization channel. The incident ray following *OP*_1_*P*_2_*Q* trajectory travels through the distortion medium first in the forward direction along **k**_1_ and then in the backward direction along **k**_2_ after the beam is backscattered by CBM. The total phase delay introduced by the distortion can be written as Δ*ψ*_1_(**k**_1_, *t*_1_) + Δ*ψ*_2_(**k**_2_, *t*_2_) where Δ*ψ_j_*(**k**, *t*) is the extra phase delay introduced by the distortion in region *j* for the beam propagating along direction **k** and passing at time *t*. The reciprocal ray *OP*_2_*P*_1_*Q* picks up a total extra phase delay Δ*ψ*_1_(−**k**_1_, *t*_3_) + Δ*ψ*_2_(−**k**_2_, *t*_4_). Their net phase difference due to the presence of the distortion medium is thus $\Delta \Psi =\underset{j=1,2}{\sum }\left[\Delta \psi_{j}\left(k_{j},t_{j}\right)-\Delta \psi_{j}\left(-k_{j},t_{j+2}\right)\right]=\underset{j=1,2}{\sum }\left[\Delta \psi_{j}\left(k_{j},t_{j}\right)-\Delta \psi_{j}\left(k_{j},t_{j+2}\right)\right]≃0$ as long as the phase distortion can be regarded as unchanged between *t_j_* and *t*_*j*+2_ and no magnetic field is present. The response time of CBM is in the order of the time lapse, *t*_*j*+2_ − *t_j_* ∼ 2(*l_t_* + *d*)/*c*, where *d* is the separation between the phase distortion region and the CBM and *c* is the speed of light. This poses an upper limit for the rate of change in the dynamic phase distortion, approaching picoseconds when the scattering medium is highly scattering and *d* tends to zero.

We have analyzed theoretically coherent backscattering mirror and shown that a multiple scattering random medium behaves as a dynamic reciprocity mirror which converts a diverging incident beam into a converging coherent backscattering one focusing on a conjugate spot in the *spatial* space. Coherent backscattering mirror is a robust phenomenon which relies on the spatial coherence of the incident beam and can also be achieved with a temporally incoherent light source. CBM is invariant with respect to an interchange of source and detector. The position of the CBM enhanced spot and the enhancement spindle does not change when the scattering medium serving as the mirror varies its position and/or orientation meeting the quasinormal incidence and emergence condition.

The CBM exhibits capabilities in compensating and correcting both static and dynamic phase distortions occurring on the optical path, attributed to the dynamic reciprocity properties of the random medium and the associated scattering matrix. It should be pointed out that although a dynamic CBM can be compared with a dynamic phase-conjugation mirror based on stimulated Brillouin scattering or four-wave mixing nonlinear mirror,^29^ CBM is fundamentally different from phase conjugation and originates from the reciprocity of light instead.

The intensity of CBM reflected light can be significantly magnified using a gain scattering medium as the mirror.^30–32^ CBM may find applications in real time correction of dynamic phase distortions (aberrations) in passive and active optical components such as correcting phase distortions in a multi-pass amplifying system. The CBM enhancement profile can be used to conveniently quantify local optical properties of a random medium in biomedical applications and remote sensing in general. The technique has also potential applications in dynamic target navigation in which CBM can be used to automatically compensate phase distortions caused by, for example, the air turbulence on the navigation laser guide beam.

## Figures and Tables

**FIG. 1.**
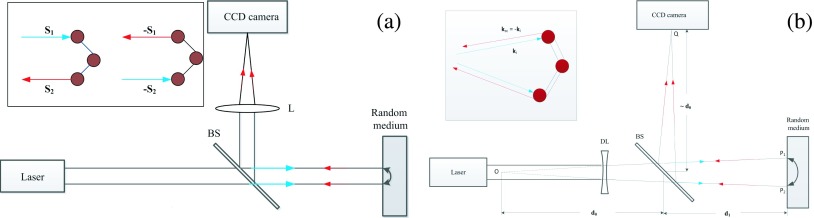
Schematic diagram for coherent enhanced backscattering (a) in the direction space and (b) in the spatial space. In (a), the angular spectrum of intensity of backscattering light is recorded by the CCD camera aided by a converging lens (L). In (b), the enhanced spot of backscattering light on the conjugate plane is recorded by the CCD camera. BS: beam splitter; DL: a diverging lens.

**FIG. 2.**
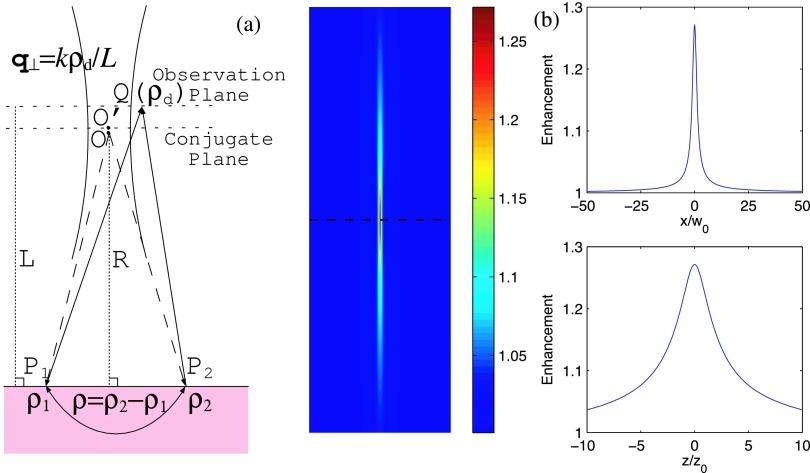
Enhancement of backscattering light in spatial space by CBM. (a) Schematic diagram. The coherent backscattering of a Gaussian incident beam in 3D forms an enhancement spindle normal to the conjugate plane. The sharpest enhancement occurs on a spot *O*′ on the conjugate plane located at *L* = *R* above the surface of the CBM. (b) Light enhancement spindle produced by a Gaussian beam (wavelength λ = 0.532*μ*m, waist *w*_0_ = 36.0*μ*m at height *Z* = 410mm, and Rayleigh range *z*_0_ = 7.57mm) reflected by a semi-infinite scattering medium (*l_t_* = 0.5mm). Horizontal and vertical profiles are displayed in the two insets. The dash lines represents the conjugate plane.

**FIG. 3.**
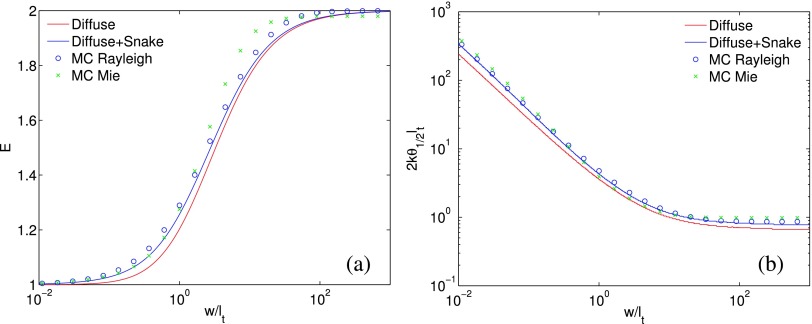
The enhancement factor (a) and the normalized FWHM (b), 2*kl_t_θ*_1/2_, of the enhanced spot on the conjugate plane computed from the diffusion approximation to radiative transfer, the diffusion approximation with snake photon correction, and Monte Carlo simulations of photons inside a semi-infinite scattering medium of Rayleigh scatterers or Mie scatterers, respectively.

**FIG. 4.**
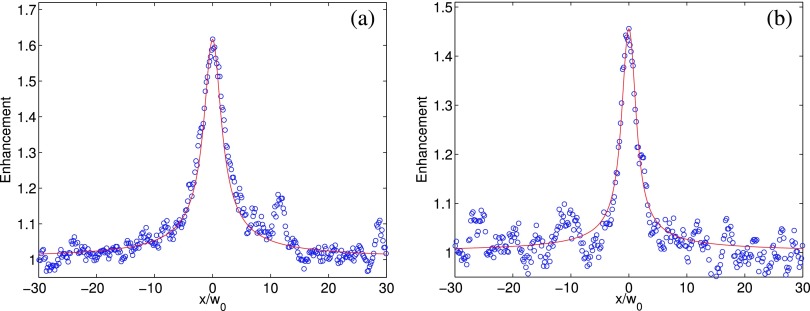
The profile of the enhanced spot on the conjugate plane produced by light backscattering from suspensions of polystyrene spheres (size: 0.49*μm*) with volume concentration of (a) 1% and (b) 0.5%. The incident beam is circularly polarized green laser (λ = 532*nm*) diverged by a concave lens (*f* = 10cm) with the waist size *w*_0_ = 36.0*μm*. The width of the beam on the sample surface is measured to be 1.93*mm*. The red solid line shows the fitting with the theoretical model with the only free parameter *l_t_*. The values of the fitted *l_t_* are 0.279*mm* and 0.534*mm*, respectively, for (a) and (b). The expected *l_t_* predicted by Mie theory is 0.270*mm* and 0.540*mm*, respectively. The speckles are more prominent in the less scattering medium (b).

**FIG. 5.**
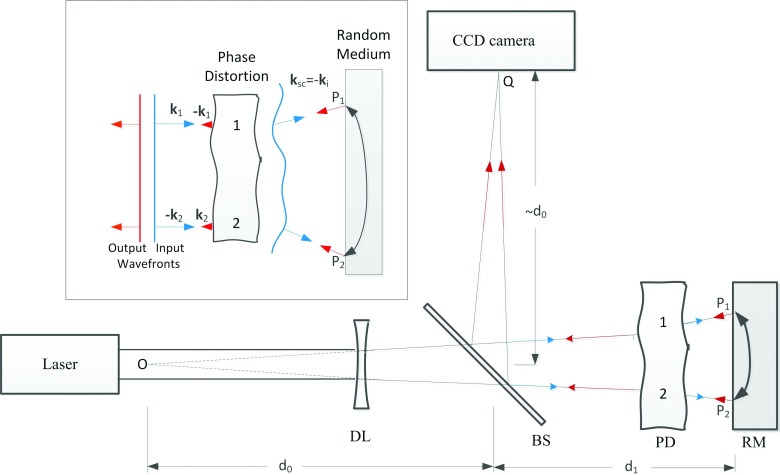
Schematic diagram of experimental setup demonstrating compensation and correction of the phase distortion between the CBM and the detector.

**FIG. 6.**
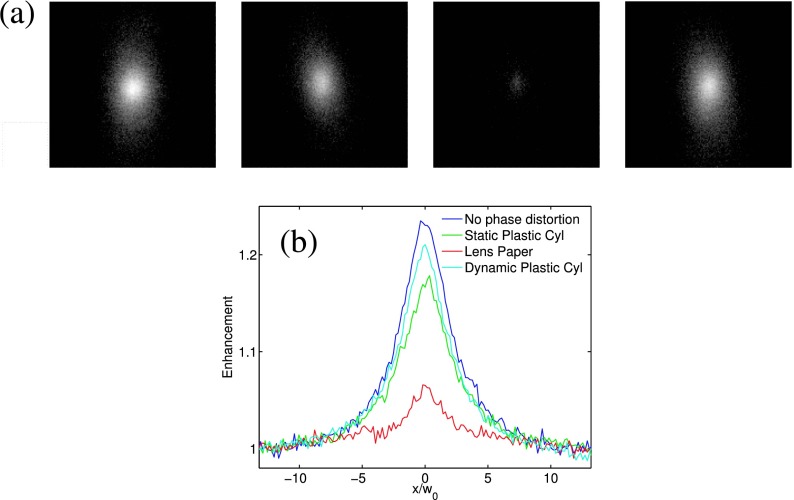
(a) The PSF images (from left to right) obtained without any phase distortion, a phase aberrator (a plastic cylinder), a thin scattering paper, and a dynamic phase aberrator (a fast moving plastic cylinder) placed in the optical path of the incident beam. The window size is 1mm × 1mm. (b) The horizontal profiles for the PSF normalized to the baseline. The light source is a supercontinuum laser (*w*_0_ = 36*μm*).
